# Single-Cell RNA-Sequencing Identifies Activation of TP53 and STAT1 Pathways in Human T Lymphocyte Subpopulations in Response to Ex Vivo Radiation Exposure

**DOI:** 10.3390/ijms20092316

**Published:** 2019-05-10

**Authors:** Maria Moreno-Villanueva, Ye Zhang, Alan Feiveson, Brandon Mistretta, Yinghong Pan, Sujash Chatterjee, Winston Wu, Ryan Clanton, Mayra Nelman-Gonzalez, Stephanie Krieger, Preethi Gunaratne, Brian Crucian, Honglu Wu

**Affiliations:** 1 NASA Johnson Space Center, Houston, TX 77058, USA; maria.moreno-villanueva@uni-konstanz.de (M.M.-V.); alan.h.feiveson@nasa.gov (A.F.); rc1025@tamu.edu (R.C.); brian.crucian-1@nasa.gov (B.C.); 2 Human Performance Research Center, University of Konstanz, 78457 Konstanz, Germany; 3 NASA Kennedy Space Center, Cape Canaveral, FL 32899, USA; ye.zhang-1@nasa.gov; 4 Department of Biology and Biochemistry, University of Houston, Houston, TX 77204, USA; branmistretta@gmail.com (B.M.); ypan6@central.uh.edu (Y.P.); ssc308@nyu.edu (S.C.); phgunara@central.uh.edu (P.G.); 5 Department of Computer Science, Johns Hopkins University, Baltimore, MD 21218, USA; wwu37@jhu.edu; 6 KBRWyle, Houston, TX 77058, USA; mayra.a.nelman@nasa.gov (M.N.-G.); stephanie.s.krieger@nasa.gov (S.K.)

**Keywords:** single-cell RNA-sequencing, radiation, human T cells

## Abstract

Detrimental health consequences from exposure to space radiation are a major concern for long-duration human exploration missions to the Moon or Mars. Cellular responses to radiation are expected to be heterogeneous for space radiation exposure, where only high-energy protons and other particles traverse a fraction of the cells. Therefore, assessing DNA damage and DNA damage response in individual cells is crucial in understanding the mechanisms by which cells respond to different particle types and energies in space. In this project, we identified a cell-specific signature for radiation response by using single-cell transcriptomics of human lymphocyte subpopulations. We investigated gene expression in individual human T lymphocytes 3 h after ex vivo exposure to 2-Gy gamma rays while using the single-cell sequencing technique (10X Genomics). In the process, RNA was isolated from ~700 irradiated and ~700 non-irradiated control cells, and then sequenced with ~50 k reads/cell. RNA in each of the cells was distinctively barcoded prior to extraction to allow for quantification for individual cells. Principal component and clustering analysis of the unique molecular identifier (UMI) counts classified the cells into three groups or sub-types, which correspond to CD4+, naïve, and CD8+/NK cells. Gene expression changes after radiation exposure were evaluated using negative binomial regression. On average, *BBC3*, *PCNA,* and other *TP53* related genes that are known to respond to radiation in human T cells showed increased activation. While most of the *TP53* responsive genes were upregulated in all groups of cells, the expressions of *IRF1*, *STAT1,* and *BATF* were only upregulated in the CD4+ and naïve groups, but were unchanged in the CD8+/NK group, which suggests that the interferon-gamma pathway does not respond to radiation in CD8+/NK cells. Thus, single-cell RNA sequencing technique was useful for simultaneously identifying the expression of a set of genes in individual cells and T lymphocyte subpopulation after gamma radiation exposure. The degree of dependence of UMI counts between pairs of upregulated genes was also evaluated to construct a similarity matrix for cluster analysis. The cluster analysis identified a group of *TP53*-responsive genes and a group of genes that are involved in the interferon gamma pathway, which demonstrate the potential of this method for identifying previously unknown groups of genes with similar expression patterns.

## 1. Introduction

In space, astronauts are constantly exposed to cosmic radiation that consists of energetic charged particles [1]. The cellular effects of space radiation exposure, particularly at the molecular level, have thus far been assessed mostly in pools of cells that have been irradiated with a single particle type [2]. However, in the space environment, only a small fraction of the cells would be traversed by particles at a given time. In such an environment, the cells may experience severe radiation damage, possibly leading to chromosomal aberrations or cell death [3,4]. Additionally, damage can be induced in bystander cells through non-targeted effect mechanisms [5,6]. Thus, it is necessary to investigate the cellular responses to space radiation exposure in individual cells in order to accurately assess the associated health risks. Radiation-induced gene expression has been assessed in populations of cultured cells or in animal tissues, while chromosome aberrations and DNA strand breaks can be analyzed in individual cells [7,8,9,10]. The single-cell RNA sequencing technique (scRNA-seq) can reveal complex regulatory relationships between genes [11], allowing us to identify pathways of distinct cell subsets in response to radiation. Scientists have been improving high throughput microfluidic techniques for the isolation of single cells for almost a decade [12]. Advancements in technology have increased the accessibility and decreased costs. Currently, single cells can be captured in an oil droplet containing one hydrogel bead (gel bead) that is labeled with unique barcoded oligonucleotides that tag individual cells [13]. scRNA-seq has been applied in multiple research fields, such as in cancer biology for exploring tumor cell heterogeneity, in immunology for identifying new T cell receptors, or in neuroscience for investigating neural cell diversity [14,15,16].

Although scRNA-seq analysis will eventually be intended for charged particles or space applications, we report the results of scRNA-seq analysis for human lymphocytes after exposure ex vivo to Cs-137 gamma rays here. Several studies reported quantitative and functional complex interactions between the different components of the immune system in response to radiation. Some of the radiation-induced late effects are related to the immune and inflammatory pathways. Furthermore, low-dose radiation exposure due to environmental sources, cosmic travel, or medical diagnostic techniques is becoming a major epidemiological concern world-wide and there is a need for understanding the mechanisms behind low-dose radiation and the potential long-term health consequences [17]. Studies regarding gene expression in human lymphocytes after gamma or X-rays irradiation have been mostly carried out in the context of developing biodosimetry techniques [18,19,20]. However, to our knowledge, no scRNA-seq studies of human blood cells in response to radiation exposure have been reported.

## 2. Results

### 2.1. Verification and Identification of Isolated Cell Subpopulations by FACS

#### 2.1.1. Cell Types

**Figure 1:**
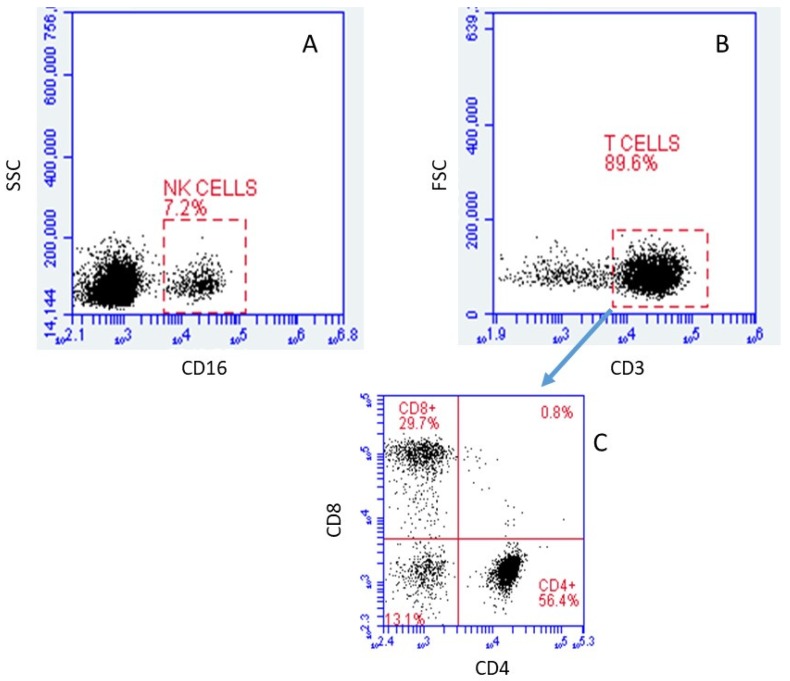
Immunophenotyping via flow cytometry analysis of cells obtained after T Cell enrichment by high affinity negative selection human T cell enrichment columns (SSC: Side scattering; FSC: Forward scattering). (**A**) Relative size vs. CD16 relative intensity, gated on CD45+. (**B**) Relative granularity vs. CD3 relative intensity, gated on lymphocytes. (**C**) CD8 vs. CD4 relative intensities, gated on T cells. Of the CD45+ population, 7.2% were positive for CD16, while 89.6% of the lymphocyte population was positive for CD3. Cells that were positive for CD4 or CD8 (but not both) accounted for 56.4% and 29.7% of CD3+ cells, respectively.

The viability of both irradiated and control cells, as determined by Guava ViaCount technology, was greater than 90%. Although we intended to only isolate T cells, some natural killer (NK) cells were included in the cell population studied. Using flow cytometry, we found that approximately 7.2% of the cells studied were NK cells (CD16+) (Figure 1A). At the same time, we found that approximately 89.6% of the cells were T-cells (CD3+) (Figure 1B). Of the CD3 positive cells, approximately 56.4% were CD4+ (CD8−), while 29.7% were CD8+ (CD4−). A fairly small fraction of cells (13.1%) were double negative in the staining, while almost none of the cells (0.8%) were double positive (Figure 1C).

#### 2.1.2. Quality of Single-Cell RNA Sequencing

The sequencing results revealed that we captured 690 control and 733 irradiated cells, as analyzed from a mean of 35 million paired-end reads per sample (Table 1). We were able to detect over 1000 genes that were associated with a cell barcode and contained at least 1 unique molecular identifier (UMI) in both samples. Most of the genes were represented by multiple UMIs illustrated by the sequencing saturation. Both of the samples reached a sequencing saturation over 80% a representation of high sequencing depth. Furthermore, both samples had greater than 97% barcode efficiency, meaning that we can confidently map these reads to individual cells and not ambient RNA or noise. The percentages of Q30 bases in barcode were 96.3% and 96.4% for the control and irradiated samples, respectively (Table 1). This is an indication that high-quality barcoded information is passing the filter and the majority of the barcodes are not being analyzed by the downstream algorithms. Figure S1A,B show further quality-control analytics. Knee plots for both the control and radiated samples show the cumulative distribution of barcodes counts vs. that of the UMI counts on a log-log scale. Barcodes that are likely associated with a cell (those that have relatively high UMI’s) are designated in green (green arrow) [21]. By contrast, the preponderance of barcodes has relatively low UMI counts and they are considered to be associated with noise. The steep drop-off (see red arrow) in the numbers of UMIs for most barcodes that represent actual cells, as opposed to barcodes that are associated with noise, is suggestive of the unbiased identification of actual cells. For both samples, the median UMI count is about 3500 for barcodes that are thought to represent actual cells. Overall, about 0.001% of the distribution of UMIs sequenced correspond to noise. 

### 2.2. Identification of Lymphocyte Subpopulations Based on scRNA-seq

**Figure 2:**
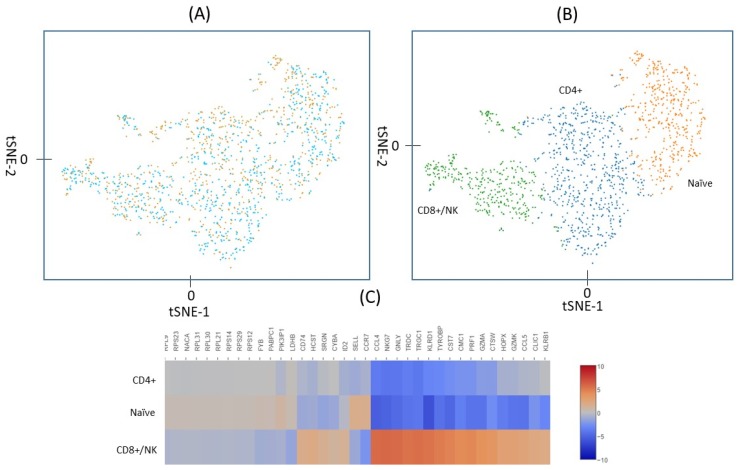
(**A**) Unsupervised t-SNE analysis of the UMI counts in the irradiated (orange) and control (blue) cells. (**B**) A graph-based hierarchical clustering algorithm separated the cells into three groups (clusters), shown as three different colors. (**C**) Heat diagram of gene expression in each of the three clusters. Marker genes identified cluster 1 (blue in **B**) as CD4+ cells, cluster 2 (orange in **B**) as naïve cells and cluster 3 (green in **B**) as CD8+/NK cells. The naïve cells include both naïve CD4+ and naïve CD8+ cells.

Of the 690 irradiated and 733 control cells, five from each treatment group had a total UMI >7000 and they were excluded from the analysis on the assumption that for these large UMI counts, more than one cell may have been captured and tagged as a single cell. The remaining 1413 cells, 685 irradiated and 728 controls, are plotted in Figure 2. Figure 2A shows the unsupervised t-Stochastic Neighbor Embedding (t-SNE) plot from the unbiased clustering of all irradiated and control cells that are based on the UMI in each of the genes in each cell. A graph-based hierarchical clustering algorithm was also used to separate the cells into three groups (three different colors) (Figure 2B). Heat diagrams, as shown in Figure 2C, were then used to identify predominant genes within each group. Cluster 3 is distinguished by expression of a set of genes, including *CCL4*, *GNLY*, *CST7*, *PRF1*, *CCL5*, which have been identified as signature genes for CD8+/NK cells [22]. Cluster 2 is characterized by the expression of *CCR7* and *SELL* that are well-known marker genes for naïve cells [23,24]. The remaining cells that did not belong to cluster 2 or 3 were then predominately CD4+ cells (cluster 1). The total cell number of the clusters 1, 2, and 3 were 603, 412, and 398, respectively. After excluding 7.2% NK cells, the ratio of CD4+ to CD8+ cells found based on UMI values was about 2:1, which is similar to the ratio that is shown in Figure 1. 

### 2.3. Gene Expression in T cells Subpopulations

**Figure 3:**
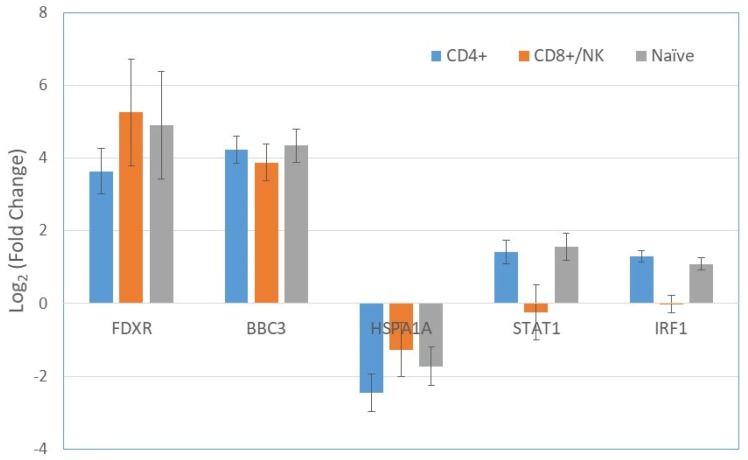
Fold changes of gene expression in selected genes after radiation exposure in the 3 clusters of CD4+, CD8+/NK, and naïve cells. *FDXR* and *BBC3* were upregulated by radiation, whereas *HSPA1A* was downregulated, in all three groups. The upregulation of *STAT1* and *IRF1* was found in CD4+ and naïve cells, but not in CD8+/NK cells. Bars represent the standard error of the mean.

Using the negative binomial regression method, the gene expression patterns of the irradiated and non-irradiated cells were compared in each of the clusters (cell subtypes) separately, as well as for all cell subtypes in combination. Generally, the expression of all genes before and after radiation exposure showed a more accurate fit to a negative binomial distribution than to a Poisson distribution. A representative example for the distribution of phosphohistidine phosphatase 1 (*PHPT1*) gene expression post irradiation is shown in Figure S2. Table 2 and Table 3 include the list of genes that were upregulated and downregulated, with a fold change threshold of 2 and *p*-values of less than 0.001 after radiation exposure. Genes that did not meet the criteria are shown in gray. Upregulated genes are mostly *TP53* related genes, including *FDXR*, *BBC3*, *CD70*, and *AEN* (Table 2). Most of the genes that were significantly upregulated in the cell population as a whole were also upregulated in one or more of the single cell subtypes. However, interestingly, *IRF1*, *STAT1*, and *BATF* were upregulated in CD4+ and naïve cells, but were unchanged in CD8+/NK cells. Among the downregulated 34 genes, *DNAJB1*, *ISG20*, *CRIP1*, *IFITM1*, and *HSPA1A* were the most remarkable (Table 3). An example of significant dysregulated genes after radiation is represented in Figure 3. *FDXR* and *BBC3* were upregulated, whereas *HSPA1A* was downregulated, in all three cell subtypes whereas *STAT1* and *IRF1* ware upregulated in CD4+ and naïve cells, but they did not change in CD8+/NK cells. 

### 2.4. Pathway Analysis

**Figure 4:**
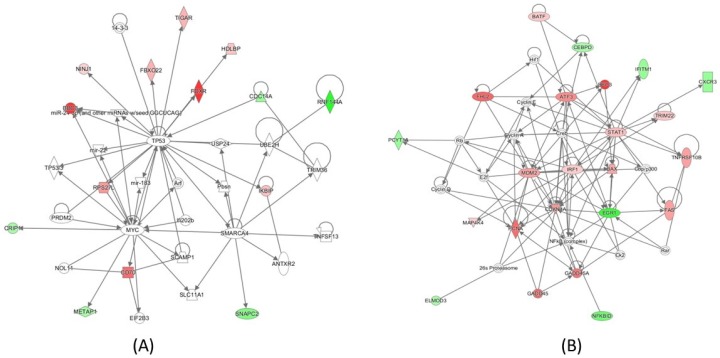
IPA analysis of the genes whose expressions were significantly altered when all cells were considered indicated that (**A**) *TP53* is the primary response to radiation exposure in human T cells after 2 Gy gamma irradiation, and (**B**) activation of the *STAT1/IRF1* signaling pathway in the irradiated cells.

Genes with significant expression changes in cells of all subpopulations combined were further studied with Ingenuity Pathway Analysis (IPA) to determine the pathways that respond to radiation exposure. As expected, radiation exposure upregulated the *TP53* response (Figure 4A). The *IRF1/STAT1* network was also activated, which involved the upregulation of genes, including *BAFT* (Figure 4B).

When pathway analysis was performed for each of the cell subtypes, *TP53* remains the top upstream regulator for all three subtypes, as determined by the *Z*-value (Table 4). Significant upstream regulators are those with a Z-value above 2. Notably, *STAT1* and *IRF1* were only upstream regulators in CD4+ and naïve cells, but not in CD8+/NK cells. These two genes, in combination with *BAFT,* which is regulated by *STAT1*, did not change expression in CD8+/NK cells (Figure 3).

### 2.5. Hierarchical Clustering

**Figure 5:**
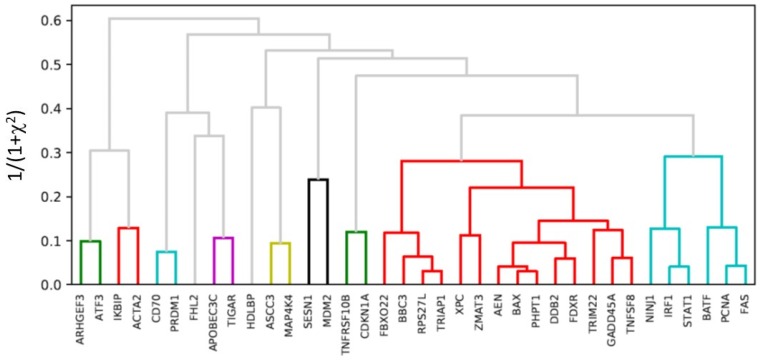
Hierarchical clustering of the radiation response genes from the expression in all of the individual cells that were irradiated. Genes that are involved in *TP53* signaling pathways (*AEN, PHPT1* and etc.) and *STAT1* signaling pathways (*IRF1, FAS, BATF, NINJ1,* and *FCNA*) are grouped together, indicating that these were the two primary pathways in response to radiation exposure in T cells.

Hierarchical clustering of the distance matrix that was obtained with bivariate negative-binomial modeling was performed to identify genes that are potentially associated with each other in response to radiation exposure. The upregulated genes were clearly separated into two clusters, one involving genes in the *TP53* network and the other in the *STAT1/IRF1* network (Figure 5). In addition, our clustering analysis showed high correlations between *CD70* and *PRDM1*, and between *ASCC3* and *MAP4K4*. The downregulated genes had low UMI counts, even in unirradiated control cells, and thus their data could not be included in the bivariate negative binomial modeling.

## 3. Discussion

Although the scRNA-seq has been used in the past to analyze human blood cells [25], most of these studies focused on identifying new types of blood cells, revealing regulatory relationships [26] or comparing transcriptomic profiles between blood and tumor cells [13]. Here, we report gene expression in individual T cells in response to ex vivo radiation exposure. Our goal was to determine whether different cells and different subtypes of T cells respond differently to radiation-induced DNA damage. Different from the study by Villani and Shekhar [25], we did not further separate CD4+ and CD8+ cells into effector memory, central memory, and effector cells. The ratio of CD4+ to CD8+ cells was about 1 in [25], which is different from the present study. However, it is known that such a ratio can vary between individuals.

In the present study, 728 cells that were irradiated with 2 Gy gamma rays and 685 non-exposed cells were analyzed for gene expression using the 10X Genomics system. Clustering analysis separated the cells into three groups that correspond to CD4+, CD8+/NK, and naïve cells, as determined by the marker genes (Figure 2). The percentages of these cell subtypes were similar to the results that were obtained from flow cytometry analysis with surface markers (Figure 1). After radiation exposure, a number of genes had significant changes in expression, as shown in Table 2. Most of these genes, including *FDXR*, *BBC3*, *PCNA*, and *GADD5A*, are known to be upregulated in blood cells after radiation exposure (Figure 3), and to be regulated by *TP53* (Figure 4A). Radiation-induced gene expression changes in peripheral blood mononuclear cells (PBMC) have been studied in the past [18] and an extended list of genes has recently been suggested as the potential biomarkers of radiation exposure [27]. Most of the genes that were upregulated in the present study have been listed as the candidate genes for potential biodosimetry applications [26]. When all subtypes of cells in the present study were considered, the fold changes in gene expressions were as high as 16 for *FDXR* and *BBC3* at three hours after radiation exposure (Table 2) [28].

Our present study demonstrated that scRNA-seq is useful for simultaneously analyzing the expression of a set of genes in different subtypes of human T cells after radiation exposure. Previous studies have reported radiation-induced changes in gene expression in subtypes of blood cells; however, each cell type was analyzed separately in these studies [29,30]. Riecke et al. [29] reported that patterns of expression of *GADD45*, *DDB2*, *CDKN1A*, *PCNA*, *BAX*, and *ATF3* genes were similar in the CD4+ and CD8+ cells after X-ray irradiation, whereas Gruel et al. [30] only reported gene expression changes in CD4+ or CD8+ cells for some of the genes. In the present study, most radiation responsive genes, such as *FDXR*, *PCNA*, and *DDB2*, were upregulated in the CD4+, CD8+/NK, and naïve cell types (Table 2), although some of the gene expression changes were not significant, as determined by the p-value criteria, because only a small number of cells were analyzed for each subtype. Our analysis also showed that *TP53* is the primary upstream regulator for all cell subtypes (Table 4). A number of genes were down-regulated after radiation exposure (Table 3). Several of those genes, including *DNAJB1, HSPA1A,* and *HSPA1B* are associated with heat shock responses. Some of the genes only showed significant downregulations when cells from all subpopulations are combined because of low numbers of cells in individual subpopulations to meet the criteria for the *p* value (Table 3). 

In contrast to the common genes upregulated in all of the cell types mentioned, we found that *IRF1*, *STAT1*, and *BATF* were upregulated in CD4+ and naïve cells, in the present study they were not upregulated in CD8+/NK cells (Table 2, Figure 3). The upregulation of these three genes indicates that the interferon gamma pathway is activated in T-cells after radiation exposure. Previous studies have shown that IRF1 and STAT1 expression is associated with radiation exposure [31]. In mouse splenocytes, after 5 Gy gamma irradiation, the phosphorylation of STAT1 turns on specific sets of IFN-gamma-inducible genes, including *IRF1* [32]. Furthermore, system biology modeling has identified *STAT1* and *IRF1* as two of the genes in a network that affect a cell’s radio-sensitivity. It is interesting to note that, in the present study, these IFN-gamma-related genes were upregulated in CD4+ and naïve cells, but not in CD8+/NK cells. One possible explanation for our observation is that CD8+/NK cells are proliferating, and are therefore more sensitive to radiation-induced apoptosis. Ozsahin et al. [33] reported that 2 Gy gamma rays induced apoptosis in 12.5% of CD4+ cells, but 20.7% of CD8+ cells. It has also been reported that *IRF1* and *STAT1* influence apoptosis in different cell types [34,35]. Further investigation is required to determine whether apoptosis influences measures of radiation-induced expression of *IRF1* and *STAT1*. The activation of STAT and TP53 signaling has also been found in response to etoposide [36,37] and several other genotoxic drugs, such as fludarabine or doxorubicin [38]. The critical importance of STAT1 is demonstrated in the mutations of the *STAT1* gene in humans. The loss of the STAT function leads to immune deficiency [39]. Some mechanisms of STAT signaling in immunity and diseases have been recently summarized [40]. There is genetic evidence that STAT1 signaling in different cell types produces antagonistic effects on innate immune response [41]. Furthermore, the expression of *STAT1* enhanced the effect of IFN-gamma and IFN-beta on the inhibition of human lung cancer cell proliferation, migration, and invasiveness [42]. The clinical benefits of TP53 have been widely described. Specifically, TP53 and other tumour-suppressor genes are emerging as potential guardians of immune integrity for the immune system [43]. 

In the present study, we also performed correlation analysis of the gene expression in response to radiation exposure between different cells (Figure 5). Such analysis has the potential to identify genes that are associated with each other. As shown in Figure 5, clustering genes that are based on the degree of correlation identified two major clusters. The first cluster contains genes were directly regulated by *TP53*, including *GADD45A*, *BAX*, *XPC*, and *BBC3*. The other cluster contains *NINJ1*, *IRF1*, *STAT1*, *BATF*, *PCNA,* and *FAS*, which are genes that are known to be associated with interferon-gamma [44]. The identification of the second cluster was also consistent with the findings that upregulation of interferon-associated genes was only found in CD4+ and naïve cells, but not in CD8+/NK cells. As expected, genes that are highly correlated have known relationships. In addition to *IRF1* and *STAT1*, Figure 5 shows high correlations between *PCNA* and *FAS*, between *RPS27L* and *TRIAP1*, and between *BAX* and *PHPT1*, among others. *STAT1* is known to regulate *IRF1*, and two genes are expected to be highly correlated [45]. However, correlations for other gene pairs are not that obvious. For instance, *TP53* has been reported to directly induce *RPS27L* that in turn promotes apoptosis [46]. On the other hand, *TRIAP1* was first characterized as a p53-inducible cell survival factor [47], and the over expression of *TRIAP1* protects cells from apoptosis that is caused by DNA damage [48]. The observation in the present study that the expressions of these two genes were highly correlated raised a question as to whether and how they regulate each other. 

One of the purposes of the present study was to determine the heterogeneity in a cell population in response to radiation exposure. As shown in Figure S2, the distribution of UMI counts in individual cells in irradiated cells can be better fitted with a negative binomial distribution than the Poisson distribution. This is also true for the distribution of UMI counts in the non-irradiated control cells. The over dispersion was partly caused due to different subpopulations of T cells in the study, in that the distribution of UMI for individual subpopulations was closer to Poisson than for all cells. However, the number of cells for individual subpopulations was low, so the results of such comparisons with the Poisson distribution were not consistent.

The present study was performed with gamma rays. Gamma-induced DNA damage and the consequent cellular responses in individual cells are expected to be similar with variations following the Poisson distribution. However, for space radiation exposure, damage in individual cells will be highly heterogeneous, as only a small fraction of the cells will be traversed by a particle of a specific type and energy at a given time. Non-targeted effects may also affect cells that are not directly traversed by the particles [6]. Therefore, gene expression in response to chronic exposures to a mixture of charged particles is expected to be found in only a fraction of cells at a given time. Damage and damage response in the human body to space radiation exposure will potentially result in health consequences, including cancer [49] and the central nervous system [50]. 

In summary, the quantification of RNA in individual T cells after radiation exposure allows determination of differential responses in different subpopulations. Out study confirmed that radiation upregulated most of the TP53 related genes. However, the expressions of *IRF1*, *STAT1,* and *BATF* were upregulated in only the CD4+ and naïve groups, but were unchanged in the CD8+/NK group, suggesting that the interferon-gamma pathway does not respond to radiation in CD8+/NK cells. 

## 4. Materials and Methods

### 4.1. Cells and Radiation Exposure

Primary human T cells were isolated from source leukocyte samples that were obtained from the Gulf Coast Regional Blood Center, Houston, Texas. To this end, peripheral blood mononuclear cells (PBMCs) from one 500 mL blood donation were separated by density-gradient centrifugation on Ficoll gradients (GE Healthcare, Life Sciences, Piscataway, NJ, USA), followed by red blood cell (RBC) lysis (Human Erythrocyte Lysing Kit, R&D Systems, Minneapolis, MN, USA). Next, the T cells were purified from the PBMC’s using high affinity negative selection human T cell enrichment columns according to the manufacturer’s protocol. These columns enabled the removal of B cells (CD19+) and monocytes (CD14+) through binding to the Ig and anti-Ig beads, resulting in a highly enriched T cell population that was collected in the eluant. The purified T cells were then resuspended in hydroxyethyl-piperazineethane-sulfonic acid buffer (HEPES) buffered Roswell Park Memorial Institute (RPMI) 1640 medium (Life Technologies/Invitrogen, Carlsbad, CA, USA) that was supplemented with 10% Fetal Bovine Serum (FBS), 1% Penicillin/Streptomycin, and 25 mM L-Glutamine (Life Technologies/Invitrogen, Carlsbad, CA). T cell purity (CD45+, CD3+) was assessed by flow cytometric analysis on a Becton Dickinson (BD) Accuri C6 for confirmation of the absence of B cells (CD19+) and monocytes (CD14+). In addition, flow cytometry analysis was also used to determine the ratio of cytotoxic (CD8+) and Helper T cells (CD4+) in the purified T cell sample.

The isolated T cells were incubated for 16 h before irradiation. All of the cells were exposed to 2 Gy gamma rays at a high dose rate at NASA Johnson Space Center, Houston, Texas, and were immediately thereafter incubated at 37 °C. The irradiator is annually calibrated with an ion chamber by the manufacture. The non-irradiated cells were kept at room temperature during the radiation time. It has been shown that some genes significantly change their expression between 2–4 h after radiation [51,52]. Therefore, after 3 h of incubation (37 °C, 95% relative humidity, and 5% CO_2_ concentration), the cells were centrifuged at 300 RCF for 5 min at 4 °C and resuspended in pre-chilled TexMacs medium (Miltenyi Biotec, Germany) containing 40% FBS (Thermal Fisher Scientific, Waltham, MA, USA) at a concentration of 2 × 10^7^ cells/mL. The cell suspension was placed on ice and resuspended in chilled 2×-freezing medium (30% Dimethyl sulfoxide, DMSO (Sigma, St Luis, MO, USA) in TexMacs medium containing 40% FCS (Thermal Fisher Scientific, Waltham, MA, USA) at a cell concentration of 1 × 10^7^ cells/mL. The cryovials were filled with 1 mL aliquots of cell suspension and then placed in a pre-cooled freezing container at −80 °C until RNA isolation procedure was performed. Cell vitality was measured while using Guava ViaCount technology (EMD Millipore, Hayward, CA, USA). All of the irradiated and controlled cells were collected in a single experiment

### 4.2. Single Cell RNA-Seq Library Preparation and Sequencing

Cell quality and viability are essential in producing reliable scRNA-seq data [53]. The process of creating a single cell suspension can stress cells and lead to cell death, which releases ambient cell-free RNA into the suspension. To avoid ambient RNA and minimize the traces of DMSO, which lowers the possible side-effects, such as changes in the transcriptome on downstream molecular assays, cell suspensions were washed 2–4 times and manually counted (using a hemocytometer) twice to assure cell viability was >90% before loading onto the Chromium platform. The libraries were created from these cells by successfully capturing cells inside gel beads in emulsion (GEM) by passing cells through a microfluidic channel. Each GEM contains a unique cellular barcode that is added to the lysed cell’s mRNA during cDNA formation by reverse transcription PCR. Library fragmentation size and quantification were measured before sequencing to ensure that the cDNA has been fragmented and barcoded correctly. The cDNA libraries were assessed while using an Agilent 2100 Bioanalyzer High-sensitivity DNA chip. For this technique, the average size of the cDNA fragments should be 500 bp and each fragment should encompass read 1 and read 2, a 16 bp 10× barcode that maps the reads back to individual cells, a 10 bp molecular identifier (UMI) that is unique for each individual read and used to quantify the transcriptome of the barcoded cell, a 8 bp i7 index read that combines four different sequences per sample to represent all four nucleotides, and Illumina paired-end constructs P5 and P7 primer sites for pair-end sequencing. Both our control and our irradiated samples had an average fragment size of 500 bp (Figure S3), so a 500 bp insert was used to count the number of fragments using a Qubit Fluorometer and by qPCR methods (data not shown). Accurate quantification allows for the proper loading of cDNA libraries for sequencing.

On the day of single-cell capture and library preparation, the control and irradiated cells were resuspended in PBS containing 0.04% bovine serum albumin (BSA) (Ambion, Foster City, CA, USA) to a final concentration of 200 cells per μL. This cell suspension was used as an input for automated single-cell capture and barcoding using the 10X Genomics Full Chromium platform. Approximately 700 single cells were captured for each sample while using the 10X Genomics Single Cell 3’ Chip (as per manufacturer recommendations Single Cell 3’ Reagent Kits v2 User Guide version CG00052) at Seq-N-Edit Core, University of Houston, Houston, Texas, USA. Single cell GEMs were generated and the single cells were uniquely barcoded. The cDNA was recovered and selected using DynaBead MyOne Silane Beads (Thermo Fisher Scientific, Carlsbad, CA, USA) and SPRIselect beads (Beckman Coulter, Brea, CA, USA). The control samples were indexed “CACTCGGA” “GCTGAATT” “TGAAGTAC” or “ATGCTCCG” and the irradiated samples were indexed “CCACTTAT” “AACTGGCG” “TTGGCATA” or “GGTAACGC”, which are Illumina sequencer compatible i7 indexes. The sequencing libraries were generated and the quality was assessed using a high-sensitivity DNA chip on 2100 BioAnalyzer (Agilent, Santa Clara, CA, USA), and the fragments were counted with Qubit Fluorometer (Thermo Fisher Scientific, Carlsbad, CA, USA) and Kapa Library Quantification Kit (Kapa Biosystems, Wilmington, MA, USA) using the AriaMX instrument (Agilent, Santa Clara, CA, USA). The libraries were sequenced using NextSeq 500 (Illumina, San Diego, CA, USA) in stand-alone mode to obtain pair end sequencing 26 bp (read1) × 98 bp (read2) and a single index 8bp in length.

### 4.3. Transcriptome Analysis

Single cell sequencing data downstream analysis was performed on the Maxwell Cluster high-performance research computing center at University of Houston, Houston, Texas, while using the analytical program, Cell Ranger 2.1.1 Single Cell Analysis Pipelines (10X Genomics, Pleasanton, CA, USA). Raw base call files that were generated by Nextseq 500 were demultiplexed using the “cellranger mkfastq” pipeline to FASTQ files. FASTQ files were aligned to the hg38 human reference genome using “cellranger count” using the STAR aligner [54]. The dimensionality of the gene-expression data was reduced to two-dimensions (2-d) using t-Stochastic Neighbor Embedding (tSNE), a nonlinear dimensionality reduction method [55]. A graph-based hierarchical clustering algorithm operating in this two-dimensional space was then used to cluster cells based on similarity of expression. These clusters were then associated with specific sub-type of T-cells (see Results) while using marker genes that were identified from the heat map of relative gene expression values [56].

### 4.4. Gene Expression Analysis in Response to Radiation

Differential gene expression in response to radiation was evaluated using a negative binomial regression model to compare the UMI distribution between the irradiated and control cells. Output from the analysis was an estimated fold change and associated *p*-value for testing the null hypothesis of no effect of radiation. This was done for all of the genes and repeated for each cluster of cells. (Note: When UMI counts for a gene consist of only 0’s and 1’s, negative binomial parameters are not estimable and Poisson regression was used instead.) These analyses were implemented while using Stata statistical software (https://www.stata.com/).

### 4.5. Pathway Analysis

Pathway analysis was performed using the Ingenuity Pathway Analysis (IPA) tool (https://analysis.ingenuity.com/pa/). For each cell cluster separately, and also for all cells combined, IPA was used to generate and rank the pathways based on the fold change and the p-values for the genes with significant expression changes.

### 4.6. Hierarchical Clustering of Genes

As a way of corroborating the IPA, we used a bivariate negative-binomial model [57] to quantify the dependence of UMI counts between each pair of genes, whose expression was significantly changed after irradiation. In addition, this analyses could identify the pairs of genes not previously known to be related, whose expression values are highly associated. More specifically, dependence was assessed in terms of a chi-squared statistic (χ2) after fitting the bivariate negative binomial regression model for each pair of genes. We then performed a non-supervised hierarchical clustering of the distance matrix with average linkage, where the distance function between the genes was defined as D = 1/(1 + χ2). Clustering was only performed on genes that were significantly upregulated. 

## Figures and Tables

**Table 1:** Quality of the single cell RNA-seq analysis.

Sequencing Parameters	Control	Irradiated
Cell Number	690	733
Total Reads	40 Million	31 Million
Genes per Cell	1131	1067
Valid Barcodes	97.8%	97.7%
Sequencing Saturation	87.1%	83.6%
Barcode Q30	96.4%	96.3%

**Table 2:** Genes whose expressions were upregulated signicantly when all of the cells or cells in individual cell types were considered. The genes meet the criteria of Fold Change (FC) > 2 (log_2_ FC > 1) and *p* < 0.001.

Gene	All Cells log_2_ FC	*p*	CD4+ log_2_ FC	*p*	CD8+/NK log_2_ FC	*p*	Naïve log_2_ FC	*p*
*FDXR*	4.05	5.9 × 10^−16^	3.63	5.2 × 10^−9^	4.52	1.7 × 10^−5^	4.90	8.9 × 10^−4^
*BBC3*	4.00	0.0 × 10^+00^	4.23	4.2 × 10^−29^	3.44	1.6 × 10^−18^	4.34	2.3 × 10^−20^
*CD70*	2.99	2.6 × 10^−9^	4.17	2.8 × 10^−6^	1.75	1.1 × 10^−2^	2.58	1.1 × 10^−1^
*AEN*	3.15	6.1 × 10^−30^	3.31	2.3 × 10^−17^	2.83	1.8 × 10^−6^	3.06	1.2 × 10^−8^
*FHL2*	3.08	5.3 × 10^−4^	2.95	7.9 × 10^−3^	2.29	1.5 × 10^−1^		
*PCNA*	2.94	0.0 × 10^+00^	3.20	6.1 × 10^−25^	3.53	2.8 × 10^−14^	2.04	2.9 × 10^−7^
*DDB2*	2.87	0.0 × 10^+00^	3.17	1.5 × 10^−21^	2.82	7.3 × 10^−11^	2.49	4.0 × 10^−12^
*GADD45A*	2.79	1.5 × 10^−15^	2.66	6.0 × 10^−9^	5.64	1.3 × 10^−4^	1.47	1.9 × 10^−2^
*ATF3*	2.33	4.8 × 10^−4^	4.20	5.6 × 10^−3^	1.78	1.2 × 10^−1^	0.77	4.5 × 10^−1^
*CDKN1A*	2.08	3.6 × 10^−4^	3.65	6.0 × 10^−4^	0.23	8.1 × 10^−1^	0.77	6.6 × 10^−1^
*TNFSF8*	2.39	1.4 × 10^−12^	2.22	2.9 × 10^−8^	2.72	4.3 × 10^−4^	3.02	5.6 × 10^−3^
*PHPT1*	2.07	0.0 × 10^+00^	2.46	7.2 × 10^−30^	1.57	6.8 × 10^−9^	1.97	3.6 × 10^−14^
*ACTA2*	2.08	6.1 × 10^−6^	1.95	1.4 × 10^−3^	2.29	3.8 × 10^−3^		
*RPS27L*	2.06	0.0 × 10^+00^	2.14	0.0 × 10^+00^	2.04	1.6 × 10^−33^	1.93	0.0 × 10^+00^
*MDM2*	1.95	7.0 × 10^−8^	1.46	1.7 × 10^−3^	4.67	1.6 × 10^−3^	1.70	1.9 × 10^−2^
*TNFRSF10B*	1.89	1.3 × 10^−5^	2.00	9.8 × 10^−4^	1.97	9.8 × 10^−2^	1.54	3.6 × 10^−2^
*FAS*	1.87	3.9 × 10^−12^	2.06	7.4 × 10^−9^	1.60	1.4 × 10^−3^	1.58	2.5 × 10^−2^
*BAX*	1.85	0.0 × 10^+00^	1.93	7.1 × 10^−37^	1.68	8.2 × 10^−16^	2.03	3.1 × 10^−21^
*ASCC3*	1.67	2.5 × 10^−9^	1.68	4.5 × 10^−5^	1.60	2.3 × 10^−3^	1.69	2.5 × 10^−03^
*PRDM1*	1.66	8.0 × 10^−4^	1.69	1.2 × 10^−2^	1.11	1.6 × 10^−1^		
*TRIAP1*	1.62	1.2 × 10^−17^	1.85	1.3 × 10^−10^	1.66	3.3 × 10^−5^	1.19	2.7 × 10^−4^
*FBXO22*	1.52	1.5 × 10^−7^	1.95	3.6 × 10^−5^	1.55	2.2 × 10^−2^	1.02	2.3 × 10^−2^
*ARHGEF3*	1.50	6.5 × 10^−9^	0.92	1.1 × 10^−2^	2.34	3.5 × 10^−4^	1.90	8.4 × 10^−5^
*TIGAR*	1.50	3.6 × 10^−5^	1.30	1.2 × 10^−2^	1.25	5.4 × 10^−2^	2.36	1.1 × 10^−2^
*STAT1*	1.32	1.2 × 10^−8^	1.42	1.3 × 10^−5^	−0.27	6.9 × 10^−1^	1.55	3.3 × 10^−5^
*APOBEC3C*	1.39	4.2 × 10^−7^	1.41	1.3 × 10^−3^	1.65	1.4 × 10^−4^	0.99	1.1 × 10^−1^
*XPC*	1.33	3.8 × 10^−14^	1.22	9.0 × 10^−7^	0.92	6.7 × 10^−3^	1.99	1.9 × 10^−7^
*SESN1*	1.30	6.6 × 10^−10^	1.66	2.1 × 10^−6^	1.11	3.3 × 10^−3^	0.95	9.7 × 10^−3^
*IKBIP*	1.26	3.2 × 10^−5^	1.58	1.1 × 10^−3^	1.23	3.6 × 10^−02^	0.97	7.5 × 10^−2^
*HDLBP*	1.18	9.2 × 10^−4^	1.25	1.7 × 10^−2^	1.55	3.9 × 10^−2^	0.77	2.4 × 10^−1^
*ZMAT3*	1.15	1.8 × 10^-04^	1.45	1.2 × 10^−03^	0.80	1.8 × 10^−1^	0.77	2.1 × 10^−1^
*TRIM22*	1.15	1.5 × 10^−10^	1.04	1.4 × 10^−5^	1.52	5.2 × 10^−4^	1.07	2.0 × 10^−3^
*BATF*	1.08	2.7 × 10^−4^	1.44	6.7 × 10^−4^	0.05	9.3 × 10^−1^	1.77	1.1 × 10^−2^
*IRF1*	1.03	1.3 × 10^−23^	1.29	2.5 × 10^−17^	0.04	8.5 × 10^−1^	1.09	1.9 × 10^−10^
*MAP4K4*	1.01	1.8 × 10^−4^	0.73	4.7 × 10^−2^	1.19	9.1 × 10^−2^	1.44	3.9 × 10^−3^
*NINJ1*	0.91	2.7 × 10^−06^	1.22	7.3 × 10^−5^	0.71	5.2 × 10^−2^	0.67	5.8 × 10^−2^

**Table 3:** Genes whose expressions were downregulated signicantly when all of the cells or cells in individual cell types were considered. The genes meet the criteria of Fold Change (FC) > 2 (log_2_ FC < −1) and *p* < 0.001.

Gene	All Cells log_2_ FC	*p*	CD4+ log_2_ FC	*p*	CD8+/NK log_2_ FC	*p*	Naïve log_2_ FC	*p*
*CRIP1*	−1.02	2.6 × 10^−9^	−1.03	1.1 × 10^−6^	−1.16	2.4 × 10^−3^	−0.64	1.1 × 10^−1^
*METAP1*	−1.05	2.2 × 10^−4^			−1.39	4.1 × 10^−2^	−1.01	3.8 × 10^−2^
*ISG20*	−1.02	5.2 × 10^−13^	−1.28	3.1 × 10^−9^	−0.84	4.5 × 10^−3^	−0.79	1.6 × 10^−3^
*DNAJB1*	−1.03	2.7 × 10^−14^	−0.61	1.0 × 10^−3^	−0.77	4.3 × 10^−3^	−1.46	9.7 × 10^−10^
*CEBPD*	−1.03	9.8 × 10^−4^	−1.69	1.4 × 10^−2^	−0.84	1.2 × 10^−2^	−2.23	1.7 × 10^−1^
*DBF4*	−1.04	9.8 × 10^−5^	−0.62	8.4 × 10^−2^	−1.96	8.2 × 10^−3^	−1.39	5.1 × 10^−3^
*ELMOD3*	−1.04	8.4 × 10^−4^	−0.91	3.9 × 10^−2^	−2.53	5.2 × 10^−3^	−0.53	3.3 × 10^−1^
*NDUFA7*	−1.05	6.7 × 10^−4^	−1.31	1.8 × 10^−2^	−0.69	1.9 × 10^−1^	−1.04	4.7 × 10^−2^
*LINC00954*	−1.07	7.7 × 10^-04^	−1.05	3.1 × 10^−2^	−1.77	6.3 × 10^−2^	−1.09	2.5 × 10^−2^
*SHOC2*	−1.10	1.8 × 10^−5^	−1.05	5.4 × 10^−3^	−1.56	8.6 × 10^−03^	−0.79	8.3 × 10^−2^
*TSEN54*	−1.09	9.8 × 10^−5^	−0.86	5.9 × 10^−2^	−1.80	5.3 × 10^−4^	−0.59	2.5 × 10^−1^
*THEMIS2*	−1.20	6.7 × 10^-04^	−1.70	6.6 × 10^−3^	−1.28	4.7 × 10^−2^	−0.37	5.6 × 10^-01^
*WDR20*	−1.13	3.3 × 10^−4^	−0.86	6.0 × 10^−2^	−1.94	1.7 × 10^−2^	−1.09	4.4 × 10^−2^
*CXCR3*	−1.17	2.0 × 10^−5^	−1.63	3.2 × 10^−4^	−0.65	6.2 × 10^−2^		
*PCYT1A*	−1.16	4.0 × 10^−4^	−1.14	1.0 × 10^−2^	−0.67	3.2 × 10^−1^	−1.81	1.5 × 10^−2^
*CCDC65*	−1.18	2.6 × 10^−4^	−1.15	1.4 × 10^−2^	−0.52	5.4 × 10^−1^	−1.55	4.2 × 10^−3^
*IFITM1*	−1.18	4.6 × 10^−7^	−1.42	8.2 × 10^−5^	−1.57	4.6 × 10^−3^	−0.70	6.5 × 10^−2^
*SF3A2*	−1.20	1.8 × 10^−4^	−1.00	3.0 × 10^−2^	−1.80	1.5 × 10^−2^	−1.15	5.4 × 10^−2^
*DOK2*	−1.23	1.5 × 10^−4^	−1.56	1.3 × 10^−3^	−0.72	2.3 × 10^−1^	−1.23	9.5 × 10^−2^
*CDC14A*	−1.23	5.8 × 10^−4^	−1.21	1.5 × 10^−2^	−0.55	4.6 × 10^−1^	−1.81	1.6 × 10^−2^
*ABHD3*	−1.22	6.9 × 10^−6^	−1.52	1.4 × 10^−4^	−0.15	8.0 × 10^−1^	−1.41	6.7 × 10^−3^
*APBB1*	−1.29	8.7 × 10^−4^	−1.97	6.9 × 10^−3^	−0.77	3.0 × 10^−1^	−1.15	5.4 × 10^−2^
*YPEL2*	−1.39	4.4 × 10^−4^	−2.63	7.3 × 10^−4^			−0.64	2.0 × 10^−1^
*RP11284N83*	−1.41	9.5 × 10^−4^	−3.11	4.2 × 10^−4^	−0.25	7.5 × 10^−1^	−0.23	7.7 × 10^−1^
*SNAPC2*	−1.48	1.4 × 10^−4^	−1.80	1.9 × 10^−3^	−1.73	3.9 × 10^−2^	−0.97	1.9 × 10^−1^
*HSPA1B*	−1.52	1.4X10^−4^	−1.63	2.3 × 10^−2^	−1.79	5.4 × 10^−2^	−1.54	3.7 × 10^−3^
*NFKBID*	−1.70	5.0 × 10^−5^	−1.51	8.4 × 10^−2^	−0.84	1.9 × 10^−1^	−3.23	2.6 × 10^−4^
*HSPA1A*	−1.76	1.4 × 10^−7^	−2.46	3.0 × 10^−6^	−1.42	5.2 × 10^−2^	−1.72	9.1 × 10^−4^
*ERI1*	−1.83	9.2 × 10^−4^	− 2.63	3.7 × 10^−3^	−0.35	7.1 × 10^−1^	−2.04	7.8 × 10^−2^
*HAUS7*	−2.05	1.8 × 10^−4^	−1.99	3.2 × 10^−3^	−1.62	3.3 × 10^−1^	−2.40	3.3 × 10^−2^
*LZTS2*	−2.20	7.5 × 10^−4^	−0.53	5.3 × 10^−1^	−3.35	2.7 × 10^−2^		
*EGR1*	−2.29	2.4 × 10^−^	−1.63	1.0 × 10^−1^	−2.84	4.9 × 10^−2^	−2.48	9.5 × 10^−3^
*GZMH*	−2.60	3.2 × 10^−4^			−2.03	5.4 × 10^−3^		

**Table 4:** *Z*-value of the upstream regulators identified in each of the three clusters generated by Ingenuity Pathway Analysis (IPA). Significantly regulated genes were defined by fold>2.0 and *p* < 0.001. STAT1 and IRF1 had a Z value below 2 in only the CD8+/NK cells.

	CD4+	CD8+/NK	Naïve
TP53	4.51	3.8	3.58
TP63	2.96	2.44	2.58
NFATC2	2.35	1.11	1.91
STAT1	2.28	0.54	2.74
IRF1	2.19	1.09	2.36
TP73	2.15	2.57	1.51
STAT5B	−2.61	−2.19	−2.22
STAT5A	−2.14	−2.19	−2.2

## Supplementary Materials

Supplementary materials can be found at https://www.mdpi.com/1422-0067/20/9/2316/s1.
