# Supplementary material for: Single-Cell RNA-Sequencing Identifies Activation of TP53 and STAT1 Pathways in Human T Lymphocyte Subpopulations in Response to Ex Vivo Radiation Exposure
Source: Int J Mol Sci. 2019 May 10;20(9):2316. doi: 10.3390/ijms20092316 (PMC6539494; doi:10.3390/ijms20092316)
Supplement: Supplementary file 1 [file ijms-20-02316-s001.pdf]

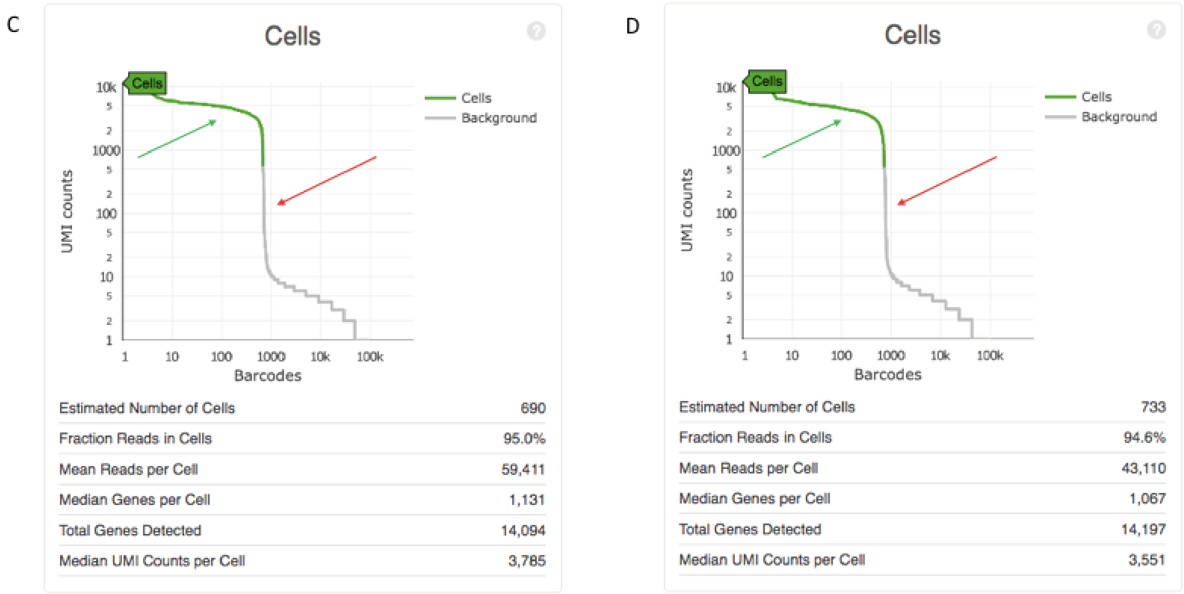

Figure S1. Knee plots of (A) control sample and (B) irradiated sample used for sequencing quality control analytics. Green arrows represents the 3500 UMI counts mapped back to cell barcodes. Red arrows represent the 0.001% of UMI counts not mapped back to cell barcodes.

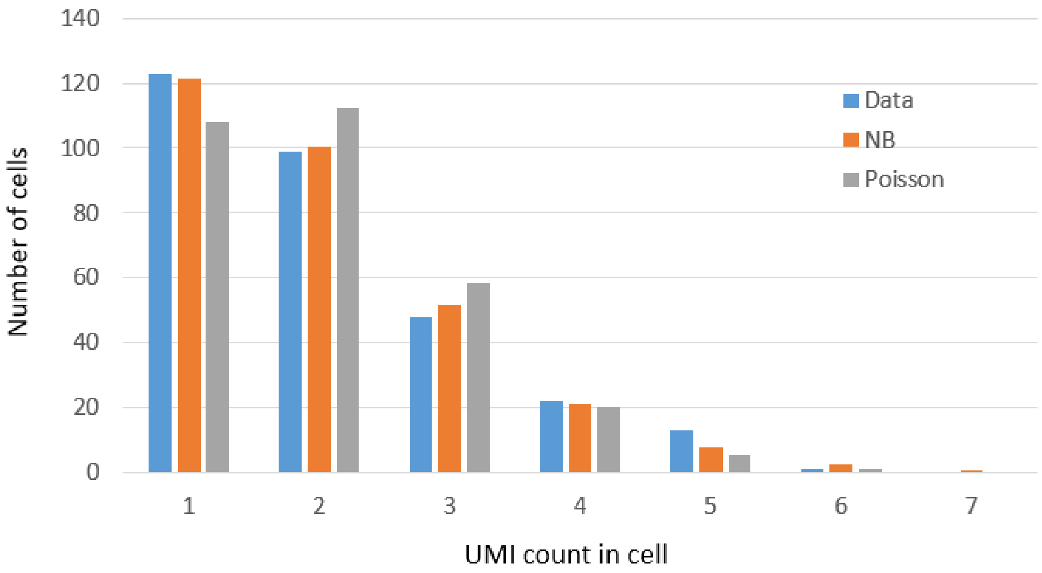

Figure S2. Distribution of UMI of PHPT1 in CD4+ cells after radiation exposure. The distribution can be fitted better with negative binomial (NB) distribution than the Poisson distribution.

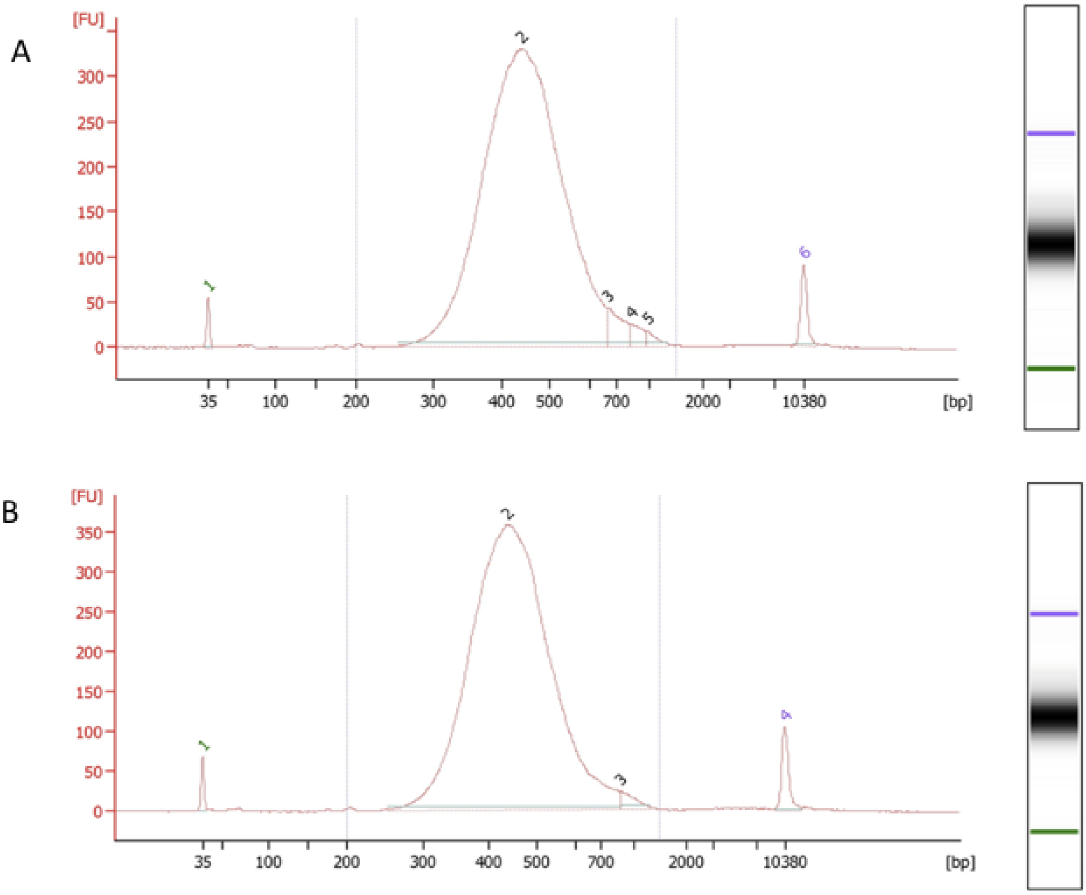

Figure S3. Average fragment size of (A) control sample and (B) irradiated sample . Average fragment size of 500bp is required for complete sequencing and identification of individual cell.
